# Cytokine-induced oxidative stress in cardiac inflammation and heart failure—how the ubiquitin proteasome system targets this vicious cycle

**DOI:** 10.3389/fphys.2013.00042

**Published:** 2013-03-06

**Authors:** Antje Voigt, Anna Rahnefeld, Peter M. Kloetzel, Elke Krüger

**Affiliations:** ^1^Institut für Biochemie, Charité-Universitätsmedizin BerlinBerlin, Germany; ^2^DZHK (German Centre for Cardiovascular Research), Partner Side BerlinBerlin, Germany

**Keywords:** proteasome, oxidative stress, inflammation, protein quality control, cytokines

## Abstract

The ubiquitin proteasome system (UPS) is critical for the regulation of many intracellular processes necessary for cell function and survival. The absolute requirement of the UPS for the maintenance of protein homeostasis and thereby for the regulation of protein quality control is reflected by the fact that deviation of proteasome function from the norm was reported in cardiovascular pathologies. Inflammation is a major factor contributing to cardiac pathology. Herein, cytokines induce protein translation and the production of free radicals, thereby challenging the cellular protein equilibrium. Here, we discuss current knowledge on the mechanisms of UPS-functional adaptation in response to oxidative stress in cardiac inflammation. The increasing pool of oxidant-damaged degradation-prone proteins in cardiac pathology accounts for the need for enhanced protein turnover by the UPS. This process is accomplished by an up-regulation of the ubiquitylation machinery and the induction of immunoproteasomes. Thereby, the inflamed heart muscle is cleared from accumulating misfolded proteins. Current advances on immunoproteasome-specific inhibitors in this field question the impact of the proteasome as a therapeutic target in heart failure.

## Introduction

### The ubiquitin proteasome system

The ubiquitin proteasome system (UPS) plays a central role in cellular protein-quality control, and MHC class I antigen presentation in viral infection. By degrading short-lived poly-ubiquitin-tagged proteins it determines the availability of regulatory proteins and controls a large number of cellular processes. This system relies on a cascade of three enzymes termed E1, E2, and E3 that conjugate poly-ubiquitin chains to specific target proteins (Ciechanover, 1994; Komander, 2009). The 26S proteasome represents the essential catalytic part of the UPS that regulates the degradation of such ubiquitin-tagged protein substrates. The standard-20S catalytic core complex is built up from 28 subunits that are arranged as four heteroheptameric rings in a α_1–7_(β_1–7_)_2_α_1–7_ structure. Within the β-rings, three standard β-subunits (β1, β2, β5) exert the catalytic activity (Groll et al., 2000). In its latent state, the α-ring is closed and may be opened upon interaction of the α-subunits with regulatory complexes like the 19S regulator, thus forming the 26S standard-proteasome (s-proteasome). The C-termini of the triple A-ATPase subunits of the 19S complex bind to pockets between the α-subunits of the catalytic core complex and thereby accomplish gate opening. Other subunits of the 19S regulator primarily recognize and bind poly-ubiquitin-chains, thus acting as the initial docking partner at the proteasome for degradation-prone proteins in the cell.

The catalytic activity of the proteasome is modulated at the level of subunit expression, subunit incorporation and by association of different regulator complexes to the proteasome core complex. In addition to s-proteasomes, mammalian cells contain a specific proteasome isoform, the so-called immunoproteasome (i-proteasome). I-proteasomes harbor alternative catalytically active β-subunits, i.e., β1i/LMP2, β2i/MECL1, and β5i/LMP7 (Aki et al., 1994). S-proteasomes are constitutively expressed in almost all non-hematopoietic cells including cardiomyocytes, endothelial cells, and fibroblasts. I-proteasomes are constitutively expressed in immune relevant cells like lymphocytes and monocytes or lymphoid tissues. Although the s-proteasome β1, β2, and β5 represent the predominant catalytic subunits in the non-stressed myocardium, mouse hearts also express i-proteasomes to a minor extent (Gomes et al., 2006). In inflammation, i-proteasomes are induced in target cells of a cytokine response, e.g., cardiomyocytes in viral cardiomyopathy (Szalay et al., 2006). Moreover, cardiac proteasomes from unchallenged hearts can consist of multiple subpopulations with different proportions of β-subunits in each β-ring (Gomes et al., 2006). Pro-inflammatory cytokines and other stress conditions also regulate the synthesis and association of the proteasome activator PA28. PA28 can interact with the α-rings of the core 20S proteasome complex. This way, the N-terminal tails of the α-subunits flip upwards, thereby facilitating substrate entry and product release through the otherwise closed gate of the 20S proteasome (Whitby et al., 2000).

### Inflammatory cytokine production and oxidative stress leading to heart failure

Inflammation and oxidative stress are both implemented in the development of acute and chronic heart failure. Oxidative stress refers to the total burden of potentially harmful reactive oxygen species (ROS) and reactive nitrogen species (RNS) that form in cellular metabolism. The most important sources of ROS/RNS include the mitochondrial electron transport chain, the nicotinamide adenine dinucleotide phosphate (NADPH) oxidase (Nox), and NO synthase (NOS) activity. Nox as the main producer of ROS in vascular cells can be induced by inflammation and a variety of cellular stress including ER stress. The Nox-isoforms Nox2 and Nox4 are expressed in cardiomyocytes, endothelial cells, fibroblasts, and inflammatory cells (Cave et al., 2006). Both, their molecular targets and their involvement in cardiac pathology have been reviewed recently (Burgoyne et al., 2012). With the exception of Nox4, the molecular chaperone Hsp90 binds to Nox proteins and regulates enzymatic activity and superoxide production (Chen et al., 2011). Disruption of Hsp90-Nox interaction and binding of Hsp70 to Nox promotes the E3-ubiquitin-ligase CHIP-dependent ubiquitylation of Nox. Thereby, Nox is transferred to proteasome-dependent degradation and ROS production is limited (Chen et al., 2012).

Cells have evolved various enzymatic and non-enzymatic defense mechanisms that directly detoxify free radicals. Whenever the demand to detoxify ROS is exhausted within a cell—a process frequently occurring in pathophysiological conditions like ischemia-reperfusion (I/R) injury—oxidative stress occurs with potential harm to the cell. Herein, the detrimental effects of ROS are particularly attributed to protein carbonylation, lipid peroxidation, and DNA damage. In addition to I/R-injury, oxidative stress is involved in a variety of other cardiovascular pathologies, such as diabetic and anthracycline-induced cardiomyopathy and cardiac hypertrophy (Khaper et al., 2010).

On the other hand, redox signaling also mediates crucial physiological processes in the heart. Likewise, a transient and self-limiting release of cytokines and chemokines in myocardial injury initiates a protective process, thereby restoring overall cardiac function. Inflammatory chemokines are mediators with multiple functions including chemoattraction of lymphocytes, natural killer cells, dendritic cells, and macrophages to the side of myocardial injury. However, pro-inflammatory cytokines may also exert detrimental effects—they contribute to heart failure in myocardial infarction and viral myocarditis. Particularly, the levels of pro-inflammatory cytokines like TNF-α and IL-6 have prognostic value for the severity of heart failure (Khaper et al., 2010). Prolonged and/or excessive cytokine/chemokine release results in immune-mediated destruction of the myocardium by infiltrating immune cells (Esfandiarei and McManus, 2008). This immune response then frequently extends to remote uninfected regions of the heart, where cytokines and inflammatory cells promote cardiac remodeling leading to heart failure. One of the basic principles of this destructive potential of a prolonged cytokine response is that inflammatory cytokines and ROS both stimulate the stress response and are re-activated by the same pathways, thereby giving rise to a vicious cycle (Khaper et al., 2010).

## Current state of the art—the UPS in protein quality control in cardiovascular inflammation

Unfolded and misfolded proteins that arise as a consequence of intrinsic or extrinsic factors like cytokine action or infection (Kruger and Kloetzel, 2012) are inherently toxic to cells (Dantuma and Lindsten, 2010). It has been shown that accumulating modified proteins tend to form high molecular weight aggregates. These aggresome-like induced structures (ALIS) act as generalized stress-induced protein storage compartments for poly-ubiquitylated defective ribosomal products (DRiPs) (Szeto et al., 2006). Chaperones like Hsc/Hsp70 bind DRiPs following their translation. In the maturation process of dendritic cells (DCs), DRiPs transiently accumulate as poly-ubiquitylated conglomerates (Lelouard et al., 2004; Rahnefeld et al., 2011). The ubiquitin ligase CHIP and the ubiquitin-domain protein BAG-1 promote substrate modification with ubiquitin. Here, CHIP and BAG-1 modulate the interplay of chaperones with the UPS, thereby facilitating proteasome degradation of ALIS in DCs (Kettern et al., 2011). ALIS need to be quickly and efficiently eliminated before they intoxicate the intracellular environment. Together with the transient protein sequestration in ALIS within the unfolded protein response, the cellular machinery is adjusted to enhance protein folding and/or to degrade misfolded proteins by the UPS. However, insufficiency of the UPS either due to ubiquitylation-deficits and/or impaired proteasome activity results in proteotoxic stress or protein toxicity. All these processes may contribute to heart failure (Powell et al., 2012).

Due to the increased demand to ubiquitylate damaged proteins to target them for proteasome destruction and the restricted levels of free ubiquitin in the cell, ubiquitin is strongly induced in the event of a proteotoxic insult (Fornace et al., 1989). Moreover, as a response of cytokine-stress or within DC maturation different enzymes of the ubiquitylation cascade are up-regulated (Ebstein et al., 2009; Seifert et al., 2010). Thereby, the efficient substrate ubiquitylation of oxidant-damaged and/or other misfolded nascent proteins in the cellular stress response ensures sufficient tagging of these degradation-prone products to be then detected by 19S subunits of the 26S proteasome. The pathophysiological impact of substrate-ubiquitylation in this matter becomes evident in neurological conformational disease. Here, the aberrant UBB+1 protein is expressed, which in high concentration is resistant to proteasome degradation causing chronic aggregation of toxic proteins (Van Leeuwen et al., 1998).

UPS dysfunction or more precisely the consequences of proteasome dysfunction are also observed in cardiomyopathies. Herein, desmin-related cardiomyopathy represents a conformational disease that is attributed to improper folding of the desmin protein. Due to unknown molecular mechanisms highly abundant misfolded desmin aggregates in the myocardium cause a functional impairment of proteasome degradation (Liu et al., 2006). A recent investigation elegantly addressed the impact of proteasome function in myocardial I/R injury making use of a heart-specific peptidase-disabled mouse β5 subunit. T60A-β5 replacement of endogenous cardiac β5 proteasome subunits reduced the chymotrypsin-like activity of the cardiac proteasome leading to pronounced structural and functional damage in I/R injury. This was attributed to increased levels of poly-ubiquitylated PTEN, which in consequence resulted in reduced Akt phosphorylation (Tian et al., 2012). In patients with dilated cardiomyopathy (DCM), proteasome functional insufficiency was also observed, here leading to the accumulation of poly-ubiquitylated, oxidant-damaged proteins in end-stage heart failure (Predmore et al., 2010).

Proteasome functional insufficiency results in a dysbalance in protein homeostasis. Here, we discuss how the proteasome responds to the need for increased protein turnover in proteotoxic stress to prevent long-term detrimental effects. It has been reported that electrophiles enhance proteasome expression through the antioxidant response elements (AREs)-Keap1-Nrf2 signaling pathway (Kwak et al., 2003). We and others have shown that mammalian cells up-regulate proteasome gene expression to compensate for proteotoxic stress caused by proteasome inhibition following the activation of the transcription factor Nrf1/TCF11 (Steffen et al., 2010). Nrf1/TCF11 and Nrf2 are members of the CNC-bZIP family both interacting with AREs within the promoters of cytoprotective genes. Both undergo regulation by the UPS—Nrf2 via ubiquitylation by KEAP1 and Nrf1/TCF11 by the ER-associated degradation pathway. An Nrf2 mediated transcriptional regulation is mainly associated with an antioxidant response toward ROS/RNS or electrophiles, whereas the TCF11 pathway seems to rely on other triggers, like proteotoxic stress (Koch et al., 2011). Also, Hsp70 recruits the chaperone-directed ubiquitin ligase CHIP to promote Nox ubiquitylation and proteasome-dependent degradation (Chen et al., 2012). Altogether, these studies provide evidence that the s-proteasome itself can ameliorate oxidative stress.

Although still a matter of controversy, several reports suggest that the 20S proteasome, which in contrast to 26S proteasome lacks ubiquitin-binding sites within the 19S cap, is capable of removing misfolded, oxidant-damaged proteins in response to oxidative stress in an ubiquitin-independent manner (Grune et al., 1997). Several lines of evidence argue against this hypothesis: (1) Most of the 26S proteasome subunits are subject to ARE-dependent transcriptional activation by Nrf1/TCF11 or Nrf2 (Kwak et al., 2003; Steffen et al., 2010); (2) Components of the ubiquitin conjugation machinery are up-regulated in response to different kinds of oxidative stress (Seifert et al., 2010; Steffen et al., 2010); and (3) Ubiquitin-rich aggregates accumulate in cells in response to oxidative stress (Szeto et al., 2006). Moreover, a large proportion of oxidant-damaged proteins is ubiquitylated and thus represents substrates of the 26S proteasome in IFN-induced oxidative stress (Seifert et al., 2010).

Another regulator of proteasome substrate turnover is PA28, which has been recently suggested to increase the ability of s- and i-proteasomes to degrade oxidant-damaged proteins (Pickering and Davies, 2012). In cardiomyocytes, PA28α overexpression resulted in increased proteasome-mediated removal of misfolded and oxidized proteins (Li et al., 2011). However, the exact function of PA28 in degradation of oxidant-damaged proteins remains to be determined.

Previous studies addressed the adaptation of the proteolytic activity of the 26S proteasome in inflammatory injuries in the heart, brain, and liver (Seifert et al., 2010; Opitz et al., 2011). In fact, prolonged sequestration of oxidant-damaged proteins in inflammation is prevented by the increased proteolytic activity of the proteasome system, which is exerted by IFN-induced formation of the i-proteasome. I-proteasomes in comparison to their s-proteasome counterpart are equipped with increased peptide-hydrolyzing activity (Sijts et al., 2000; Strehl et al., 2006; Voigt et al., 2010) and more efficient degradation capacity of ubiquitylated proteins. The effective removal of oxidant-damaged toxic proteins as a consequence of i-proteasome-function not only guarantees a steady state in protein metabolism, but also ensures cell viability in cellular stress (Seifert et al., 2010). Likewise, i-proteasomes are perfectly suited to prevent the consequences of aggravated inflammatory injury of the myocardium in enteroviral cardiomyopathy. Here, cytokine response and cytopathic effects of viral infection challenge the cellular unfolded protein response in cardiomyocytes. As a consequence of its superior proteolytic capacity, the i-proteasome eliminates toxic protein aggregates in the heart and in this way preserves cell viability and tissue integrity in cardiac inflammation (Opitz et al., 2011).

## Future perspectives

Recent studies suggest that the heart possesses an innate immune system that is intended to delimit tissue injury and regulate homeostatic responses (Mann, 2011). Toll-like receptors (TLR) and Nucleotide-binding oligomerization domain-containing protein-like receptors (NLR) act as pattern recognition receptors (PRRs) that bind conserved motifs of pathogens. TLRs and NLRs activate distinct signaling pathways, which promote the activation of transcription factors NFκ B and IRF3 to induce inflammatory cytokines, type I interferons (IFN) and chemokines (Kawai and Akira, 2011). Recent studies demonstrate that TLR and NLR also recognize molecular patterns of endogenous material, so called alarmins belonging to the family of damage-associated molecular patterns (DAMPs) (Liu et al., 2009). Alarmins are constitutively expressed and released upon myocardial damage such as myocardial ischemia or viral myocarditis. Upon activation of TLRs and NLRs these DAMPs recruit phagocytes to remove cell debris and microbes to restore tissues homeostasis.

**Figure 1:**
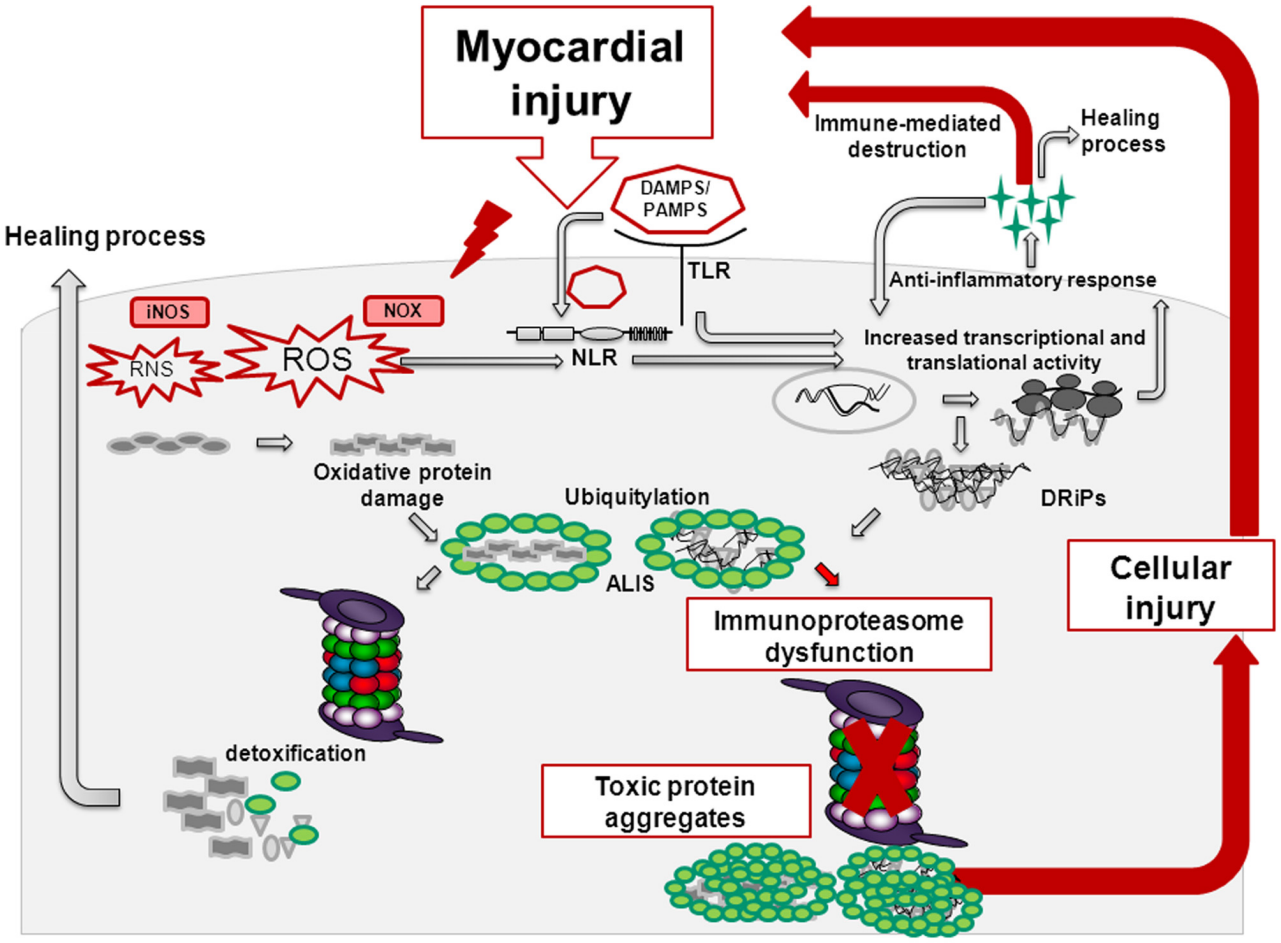
**Myocardial injury induces reactive oxygen species (ROS) and reactive nitrogen species (RNS) via the activity of NADPH-oxidase (NOX) and nitric oxide synthase (NOS).** Thereby, endogenous material being referred to as danger-associated molecular patterns (DAMPs) is released. DAMPs activate membrane-bound Toll-like receptors (TLRs) and cytoplasmatic Nucleotide-binding/oligomerization domain-like receptors (NLRs). TLR and NLR-signaling results in cytokine and chemokine release. Chemokines attract inflammatory cells and thereby facilitate tissue repair. Upon activation of the mammalian target of rapamycin (mTOR)-pathway, cytokines activate the cellular translation machinery giving rise to an increased pool of misfolded, damaged proteins known as defective ribosomal products (DRIPs). These nascent proteins are prone to be further modified by ROS/RNS eventually leading to an imbalance in the protein homeostasis with a surplus of misfolded, oxidant-damaged proteins in the cell. Here, regulatory components within the proteasomes take action—in inflammation, immunoproteasomes with increased proteolytic activity are formed that ensure the timely degradation of these inherently toxic protein aggregates. However, whenever the immunoproteasome is dysfunctional or not properly assembled, proteotoxic aggregates accumulate thereby promoting cell death. This in turn creates a vicious cycle eventually potentiating cardiac remodeling and heart failure.

Whereas short-term TLR/NLR activation confers protective effects in the injured heart, prolonged, and/or aggravated PAMP/DAMP-signaling results in the overwhelming recruitment of inflammatory cells, thereby promoting apoptosis, cardiac remodeling, and heart failure (Figure 1) (Fernandez-Velasco et al., 2012). TLR-activation also triggers the formation of ROS/RNS, which upon damage of proteins, lipids and DNA generate oxidation-specific epitopes. These molecular patterns represent targets of PRRs and further potentiate the inflammatory response (Miller et al., 2011). In acute cardiac injury e.g., in acute enteroviral myocarditis, formation of the i-proteasome in cardiac cells and constitutive i-proteasome expression in invading inflammatory cells protect these cells from cellular death. Here, the consequences of inflammation-induced ROS leading to oxidant protein damage are counterbalanced by the increased protein turnover rate of i-proteasomes. One downstream target of oxidant-damage in cytokine stress is Iκ Bα (Seifert et al., 2010). NFκ B-signaling relies on the proteasome-dependent degradation of Iκ Bα, a process that is clearly accelerated by i-proteasomes in comparison to their s-proteasome counterparts (Visekruna et al., 2009; Opitz et al., 2011). With the preservation of cellular integrity in cardiac inflammation, i-proteasome function may limit the liberation of alarmins that could exacerbate inflammatory responses in the injured heart.

This physiological adaptation of proteasome function as observed in viral myocarditis (Opitz et al., 2011) and experimental acute encephalomyelitis (EAE) (Seifert et al., 2010) is challenged by the fact that inhibition of i-proteasome activity by specific small molecular compounds (Huber et al., 2012) or gene-deletion of the i-proteasome in mice severely attenuates inflammation in autoimmune models of rheumatoid arthritis and inflammatory colitis (Muchamuel et al., 2009; Basler et al., 2010; Schmidt et al., 2010). Here, i-proteasome dysfunction suppresses Th1 and Th17, but enhances regulatory T cell differentiation, thereby limiting autodestruction by inflammatory cells (Kalim et al., 2012). Disease attenuation upon i-proteasome inhibition was also attributed to reduced pro-inflammatory cytokine levels at the side of the injury. Thereby, DAMP-signaling and oxidative stress are actually prevented here at very early stages, which in turn accounts for a steady-state in protein metabolism and reduced recruitment of inflammatory cells (Figure 1). Similar to these different reports on i-proteasome function in autoimmunity, experimental studies on first-generation proteasome inhibitors in atherosclerosis (Yu and Kem, 2010) and myocardial ischemia (Powell et al., 2012) yielded conflicting results. There is general agreement that proteasome inhibitors may be “poisons or remedies” (Meiners et al., 2008). With the second-generation of subunit-specific proteasome inhibitors it remains critical, but is conceivable that the anti-inflammatory effects of these compounds may be valuable in cardiovascular injury as well. With the aim to identify these cardiac inflammatory conditions that may benefit from i-proteasome-specific inhibitors, further research should envisage the effects of i-proteasome function in atherosclerosis, in I/R injury, cardiac hypertrophy, autoimmune myocarditis, and DCM.

## Funding

This study was supported by the Deutsche Forschungsgemeinschaft: DFG SFBTR 19 B3 and DFG VO 1602/1-1; SFB 740 B3 and KR 1915/5-1 to Elke Krüger.

### Conflict of interest statement

The authors declare that the research was conducted in the absence of any commercial or financial relationships that could be construed as a potential conflict of interest.
